# The relationship of carotid intima-media thickness with anthropometric and metabolic parameters in patients with classic congenital adrenal hyperplasia

**DOI:** 10.3906/sag-2001-57

**Published:** 2021-08-30

**Authors:** Hale TUHAN, Tülay ÖZTÜRK, Gönül ÇATLI, Sezer ACAR, Ayhan ABACI, Tuğba EGELİ, Korcan DEMİR, Şule CAN, Handan GÜLERYÜZ, Bumin DÜNDAR, Ece BÖBER

**Affiliations:** 1 Division of Pediatric Endocrinology, Department of Pediatrics, Dokuz Eylül University, Faculty of Medicine İzmir Turkey; 2 Division of Pediatric Radiology, Department of Radiology, Dokuz Eylül University, Faculty of Medicine İzmir Turkey; 3 Division of Pediatric Endocrinology, Department of Pediatrics, Izmir Katip Celebi University, Faculty of Medicine, İzmir Turkey; 4 Division of Pediatric Endocrinology, Department of Pediatrics, Tepecik Training and Research Hospital, İzmir Turkey

**Keywords:** Congenital adrenal hyperplasia, carotid intima-media thickness, subclinical atherosclerosis

## Abstract

**Background/aim:**

We aimed to determine the presence of subclinical atherosclerosis using carotid intima-media thickness (CIMT) and biochemical parameters in children and adolescents with congenital adrenal hyperplasia (CAH).

**Materials and methods:**

Thirty-four patients with classic congenital adrenal hyperplasia due to 21-hydroxylase deficiency on regular glucocorticoid treatment for ≥3 years and 31 healthy subjects were included in the study**.** The patients were divided into two groups according to the degree of control of the clinic, laboratory, and radiological parameters as a) “uncontrolled” [n= 22; with increased height velocity (HV) standard deviation score (SDS) (≥2 SDS), advanced bone age, serum 17-OH progesterone <2.0 and ≥10.0 ng/mL or androstenedione <0.3 and ≥ 3.0 ng/mL] or b) “controlled” [n= 12; with HV SDS < 2, bone age (BA)/ chronologic age (CA) ratio < 1.2, serum 17-OH progesterone between 2 and 10 ng/mL and androstenedione between 0.3 and 3.0 ng/mL]. Ultrasonographic examination of carotid artery was performed by the same radiologist using a B-mode ultrasound system.

**Results:**

There was no significant difference between the CAH and control groups in terms of median (IQR) CIMT values [0.47 (0.05) mm and 0.47 (0.07) mm, respectively; p > 0.05]. When subgroup comparisons were done in terms of median (IQR) CIMT values, there was no significant difference among the controlled, uncontrolled, and healthy control groups [0.45 (0.03) mm, 0.47 (0.04) mm, 0.47 (0.07) mm, respectively; p> 0.05]. In addition, CIMT levels were similar according to sex and disease control status.

**Conclusion:**

In this study, the CIMT values of CAH cases were similar to those of healthy subjects.

## 1. Introduction

Congenital adrenal hyperplasia (CAH) is a group of autosomal recessive disorders caused by mutations in genes encoding the enzymes that are responsible for the biosynthesis of cortisol. Deficiency of 21-hydroxylase (21-OH) enzyme is the most common type of CAH, accounting for >%95 of all CAH cases [1]. This type of CAH is characterized by a decrease in the production of cortisol and/or aldosterone and the accumulation of precursor steroid hormones in the steroidogenic pathway [2]. This disorder has a quite broad spectrum of clinic form and is classified as classical type [salt-wasting (SW) and simple virilizing (SV)] and mild nonclassic or late-onset CAH according to the degree of enzyme deficiency. The SW type has the most severe phenotype and accounts for 75% of the classic type. The SW type, usually with <2% residual enzyme activity of 21-hydroxylase, is presented by insufficient cortisol and aldosterone synthesis, and excess androgen production. In SV type, unlike SW, mineralocorticoid synthesis is sufficient. The nonclassic type is characterized by an overproduction of androgens only in adulthood [2,3]. The main goal in the treatment of the disorder is to replace the hormones that are deficient (cortisol and/or mineralocorticoid) and to suppress the androgen hormones that are increased excessively [3]. For this purpose, in clinical practice, hydrocortisone and fludrocortisone are frequently used for treatment.

Congenital adrenal hyperplasia due to 21-OH deficiency is often associated with risk of obesity, insulin resistance, hypertension, hyperandrogenism, and dyslipidemia among children and adolescents [4,5]. Intermittent hypercortisolism associated with treatment and adrenomedullary dysfunction lead to these metabolic disorders, resulting in an increased risk of developing metabolic syndrome and atherosclerosis in CAH [6]. Although various organizations have different approaches in defining metabolic syndrome, it is generally defined as the presence of three of the following five criteria; increased waist circumference, glucose intolerance, high triglyceride, low high-density lipoprotein cholesterol, and hypertension [7]. Metabolic syndrome components that occur in this disease may cause endothelial dysfunction and predispose patients to coronary artery disease and stroke at an early age [6]. Carotid intima-media thickness (CIMT) is a noninvasive test that is widely used to define early subclinical atherosclerosis. Increased CIMT is a parameter to predict increased risk of cardiovascular events such as coronary artery disease and stroke [8]. Increased CIMT has previously been reported in children with obesity, type 1 diabetes mellitus and hyperlipidemia [9,10]. However, there are only a limited number of studies evaluating CIMT in children and adolescents with CAH [11–15].

The present study aimed to determine the presence of subclinical atherosclerosis using CIMT and biochemical parameters in children and adolescents with CAH.

## 2. Materials and methods

### 2.1. Subjects

This prospective study was conducted in 2014–2015, at Dokuz Eylül University in İzmir. Thirty-four patients with classic congenital adrenal hyperplasia due to 21-hydroxylase deficiency and 31 healthy subjects were included in the study. Age, pubertal status, and sex-matched healthy controls were recruited from the general pediatric outpatient clinic. Those with chronic diseases were excluded from both CAH and the control groups. The inclusion criteria for the CAH patients were as follows i: having regular follow-up every 3 months after diagnosis at our institution, ii: using hydrocortisone ± fludrocortisone treatment for at least 3 years. In this study, which included classical type CAH cases, SW and SV classification was determined according to clinical and laboratory findings. Accordingly, patients who presented with severe cases of salt wasting (vomiting, failure to gain weight, lethargy, hyponatremia, hyperkalemia, and acidosis) in the early period (2 weeks–1 month) were evaluated as SW, and who were admitted with signs of androgen excess (cliteromegaly, penile enlargement, rapid growth, advancement in bone age, premature development of pubic and/or axillary hair) at a later age (2–3 years) and without salt loss were evaluated as SV [1–3]. 

The children’s legal guardian(s) provided written informed consents. The study was conducted according to the Declaration of Helsinki, and the study protocol was approved by the local ethics committee.

### 2.2. Methods

Demographic and clinical data including chronological age (CA), sex, type of CAH, doses of hydrocortisone (mg/m^2^/day) and fludrocortisone (mg/m^2^/day), weight standard deviation score (SDS), height, height SDS, body mass index (BMI) SDS, height velocity (HV), height velocity SDS, blood pressure (BP), and pubertal status (as prepubertal or pubertal) were collected. Measurement of height, weight, blood pressure, and bone age (BA) evaluation are stated in the following source [16]. The auxological evaluation was done according to Turkish National Growth Charts [17]. Blood pressure SDS was calculated according to Child Metrics www.ceddcozum.com, www.childmetrics.org) [18]. Obesity was defined as a BMI at or above the 95th percentile of children of the same age and sex.

 After an overnight fast, serum total cholesterol (TC), high-density lipoprotein (HDL)-C, low-density lipoprotein (LDL)-C and triglycerides (TG), fasting blood glucose (FBG), 17-OH progesterone and androstenedione were taken between 08:00 and 09:00 from all CAH patients. Androstenedione and 17-OH progesterone measurements were performed with commercially available radioimmunoassay kits. 

The patients were divided into two groups according to the degree of control of clinic and laboratory parameters as a) “uncontrolled” [n= 22; with increased HV SDS (≥2 SDS), advanced bone age, serum 17-OH progesterone <2.0 and ≥10.0 ng/mL or androstenedione <0.3 and ≥ 3.0 ng/mL] or b) “controlled” [n= 12; with HV SDS < 2, bone age (BA)/chronologic age (CA) ratio < 1.2, serum 17-OH progesterone between 2 and 10 ng/mL and androstenedione between 0.3 and 3.0 ng/mL]. 

### 2.3. Imaging

Ultrasonographic examination of carotid artery was performed using a B-mode ultrasound system with a high-definition L12-5 linear wide band transducer (Philips HDI 5000 System, Bothell, WA, USA). Both left and right common carotid arteries (CCA) were scanned. The longitudinal plane images of the CCA at 1–2 cm proximal to the carotid bulb were obtained and digitally archived. All ultrasound examinations were performed according to a standardized protocol by the same radiologist (TO) who was blinded to the clinical details of the participants. Archived images were transferred to a workstation, and intima-media thickness (IMT) values were automatically measured using a computer software (Q-LAB, ATL-Philips, Bothell, WA, USA). The measurements were performed retrospectively in a single-blind manner. The mean left and right CCA measurements were obtained, and the mean IMT values were recorded. 

A radiograph of the left hand was used to determine BA using the Greulich-Pyle atlas. All bone ages were estimated by the same endocrinologist (HT). BA/CA rates greater than 1.2 were defined as advanced bone age.

## 3. Statistical analysis

Statistical analyses of the data were conducted using SPSS 21.0 (SPSS 21.0 for Windows, IBM Corp., Armonk, NY, USA). Distribution of data was evaluated with the Kolmogorov–Smirnov test. For numerical comparisons between the study groups, the data was compared using Student’s t-test (for normally distributed data) and the Mann–Whitney U test (for nonnormally distributed data). Pearson’s chi-squared test was used for the comparison of categorical variables. The Kruskal–Wallis test was used to compare CIMT differences between more than two groups [(controlled and uncontrolled CAH patients and healthy control cases) (SW type, SV type, and healthy control)]. Spearman’s or Pearson’s correlation analysis was used to investigate the association between two variables. Data are expressed as mean ± standard deviation (SD) or median (IQR). p < 0.05 was considered statistically significant.

## 4. Results 

The clinical and laboratory characteristics of the children and adolescents are summarized in Table 1. A total of 34 patients with CAH (mean age of 10.4 ± 3.9 years, 17 males, 16 prepubertal) and 31 healthy subjects (mean age of 10.8 ± 3.6 years, 16 males, 11 prepubertal) were included in the study. The SW type of CAH represented 64.7% (n= 22) of the studied patients, while the SV type represented 35.3% (n= 12). All patients were receiving glucocorticoid substitution therapy with hydrocortisone; in addition, 22 were receiving fludrocortisone. None of the patients was receiving any additional medication.

**Table 1:** Clinical and laboratory characteristics of the study groups.

	All cases			Male			Female		
	CAH group(n=34)	Control group(n=31)	p	CAH group(n=17)	Control group(n=16)	p	CAH group(n=17)	Control group(n=15)	p
Age (years)	10.4 ± 3.9	10.8 ± 3.6	0.603a	9.1 ± 3.4	10.1 ± 3.3	0.351a	11.7 ± 4.1	11.7 ± 3.8	0.974a
Age at diagnosis (years)	0.14 (2.8)	-	-	0.2 (2.7)	-	-	0.02 (5.80)	-	-
Duration of treatment (years)	8.5 ± 3.7	-	-	7.8 ± 3.4	-	-	9.1 ± 3.9	-	-
Sex (M/F)	17 / 17	16 / 15	0.897c	-	-	-	-	-	-
Prepubertal/pubertal	16/ 18	11 / 20		11/8	7/9		7/10	4/11	
Pubertal stage 2	6	7		3	3		3	4	
Pubertal stage 3	3	4	0.157c	1	2	0.227c	2	2	0.388c
Pubertal stage 4	2	3		1	2		1	1	
Pubertal stage 5	7	6		3	2		4	4	
Normal/overweight/obese (n)	15/8/11	28/1/2	<0.001c	9/3/6	14/1/1	0.644c	6/5/5	14/0/1	0.616c
Weight SDS	0.63 ± 1.20	0.21± 0.93	0.128a	0.5 ± 1.4	0.2 ± 0.9	0.528a	0.8 ± 1.0	0.2 ± 1.0	0.110a
Height SDS	–0.17 ± 1.42	0.63 ± 1.05	0.012a	0.2 ± 1.5	0.6 ± 0.9	0.391a	–0.6 ± 1.1	0.7 ± 1.2	0.004a
BMI SDS	1.21 ± 0.72	–0.34 ± 1.3	< 0.001a	1.1 ± 0.8	–0.1 ± 1.0	0.001a	1.3 ± 0.6	–0.1 ± 1.0	<0.001a
SBP (mmHg)	102 ± 12	104 ± 11	0.458a	106 ± 11	107 ± 14	0.871a	98 ± 13	102 ± 7	0.389a
SBP SDS	0.14 (1.8)	–0.42 (0.9)	0.204b	0.50 (1.36)	–0.25 (0.75)	0.076b	–0.13 ± 0.96	–0.12 ± 0.93	0.993a
DBP (mmHg)	63 ± 11	68 ± 8	0.101a	67 ± 12	71 ± 11	0.376a	60 ± 11	66 ± 5	0.148a
DBP SDS	0.32 ± 0.9	0.48 ± 0.6	0.495a	0.53 ± 0.84	0.41 ± 0.38	0.670a	0.15 ± 0.98	0.51 ± 0.75	0.291a
FBG (mg/dL)	84.6 ± 9.3	88.9 ± 9.0	0.086a	84 (17.5)	89.5 (17)	0.024b	83.7 (11)	88.0 (10)	0.041b
HDL (mg/dL)	57.2 ± 12.6	52.1 ± 9.6	0.112a	61.8 ± 12.4	54.9 ± 10.6	0.142a	56.1 ± 12.9	49.2 ± 8.1	0.586a
LDL (mg/dL)	93.0 (46.7)	94.5 (32.0)	0.788b	104.5 ± 29.0	88.6 ± 26.1	0.153a	95.5 ± 31.9	102.2 ± 31.0	0.130a
TC (mg/dL)	175.1 ± 33.1	161.5 ± 33.1	0.140a	180.3 ± 33.1	157.3 ± 31.2	0.078a	169.7 ± 33.2	165.6 ± 35.8	0.758a
TG (mg/dL)	75 (41.5)	79 (49.2)	0.563b	74 (37)	68 (36)	0.817b	83 (63)	102 (49)	0.853b
Hydrocortisone dose (mg/m2/day)	12.8 ± 4.1	-	-	13.2 ± 3.7	-	-	12.4 ± 4.5	-	-
Fludrocortisone treatment (n)	22	-	-	12	-	-	10	-	-
Fludrocortisone (mg/day)	0.1 (0.0)	-	-	0.1 (0.0)	-	-	0.1 (0.0)	-	-
Fludrocortisone(mg/m2/day)	0.09 (0.08)			0.10 (0.15)			0.08 (0.05)		
CIMT (mm)	0.47 (0.05)	0.47 (0.07)	0.692b	0.48 (0.08)	0.49 (0.08)	0.929b	0.47 (0.03)	0.46 (0.04)	0.502b

aStudent’s t-test, bMann–Whitney U test, cChi-squared test. The values are presented as mean ± SD or median (IQR).BMI-SDS: body mass index standard deviation score of body mass index, SBP: Systolic blood pressure, DBP: Diastolic blood pressure, HDL: High-density lipoprotein, LDL: Low-density lipoprotein, TC: total cholesterol, TG: triglyceride, FBG: Fasting blood glucose, CIMT: Carotid intima-media thickness

CAH patients had significantly lower height SDS and significantly higher BMI SDS compared to healthy subjects (p = 0.012, p < 0.001, respectively). No significant difference was detected between CAH patients and healthy subjects in terms of age, sex, puberty, weight SDS, systolic blood pressure (SBP) SDS, diastolic blood pressure (DBP) SDS, FBG, HDL, LDL, TC or TG (p > 0.05). None of the children in the CAH and control groups had hypertension. Eleven (32.3%) patients in the CAH group were obese. Median age at diagnosis of CAH was 0.14 years (IQR, 2.8). Mean duration of the treatment was 8.5 ± 3.7 years. While the median age of diagnosis of SW patients was 0.04 years (IQR, 0.15), the median age of diagnosis of SV patients was 6.0 years (IQR, 4.9). Mean serum 17-OH progesterone and androstenedione concentrations in the CAH group were 13.58 ± 12.85 ng/mL and 2.88 ± 3.44 ng/mL, respectively. Median (IQR) CIMT values of CAH and control groups were 0.47 (0.05) mm and 0.47 (0.07) mm, respectively. There was no significant difference between the CAH and control groups in terms of CIMT values (p = 0.692). When compared according to sex, males with CAH had significantly higher BMI SDS than healthy males, and females with CAH had significantly higher BMI SDS and height SDS than healthy females (p < 0.05); however, there was no difference in terms of CIMT values (p > 0.05) (Table 1). 

**Figure 1:**
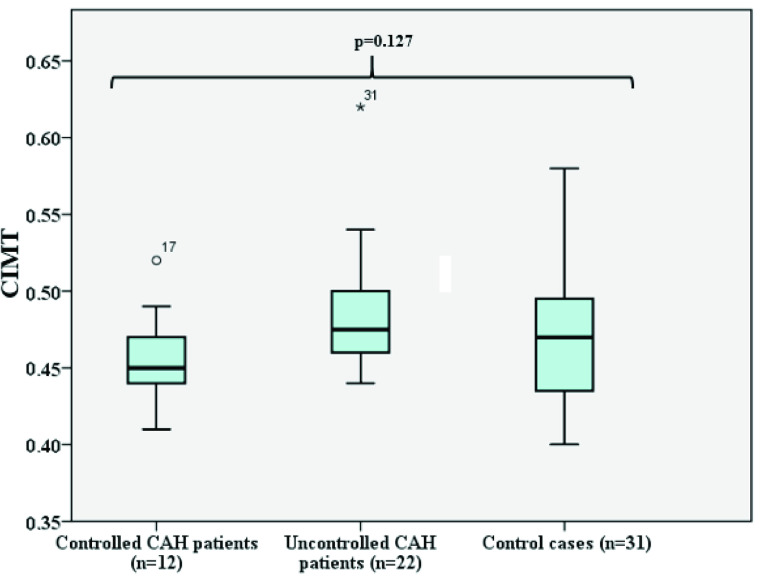
Comparisons of CIMT values according to status of disease control.

**Figure 2:**
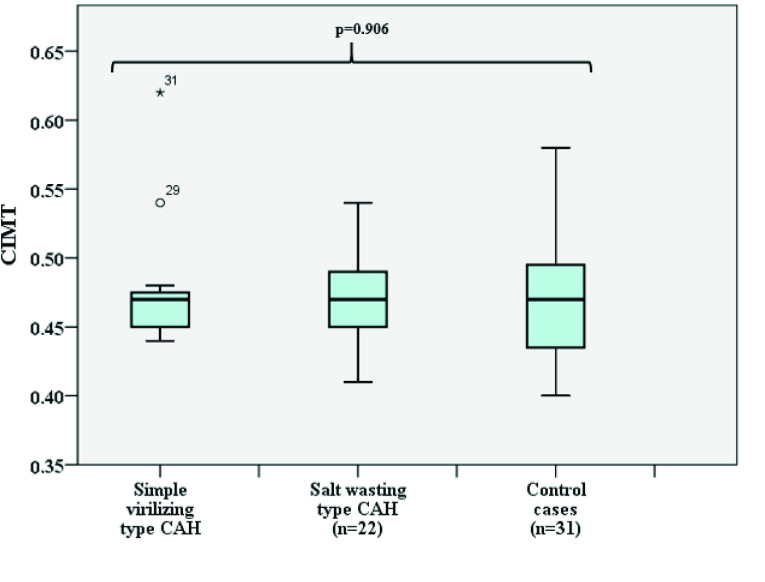
Comparisons of CIMT values in terms of CAH types.

When we compared the clinical and laboratory characteristics of CAH patients according to metabolic control, no difference was found in terms of age, age at diagnosis, sex, puberty, duration of treatment, hydrocortisone dose, fludrocortisone dose, weight SDS, height SDS, BMI SDS, SBP SDS, DBP SDS, FBG, HDL, LDL, TC, TG, HV SDS, or BA/CA ratio (p > 0.05) (Table 2). When subgroup comparisons were done in terms of CIMT values, there was no significant difference between the controlled, uncontrolled, and healthy control groups (p = 0.127, Table 2, Figure 1). In addition, there was no significant differences between the SW type CAH, SV type CAH, and healthy control groups in terms of CIMT values, SBP SDS, and DBP SDS (p = 0.906, Figure 2, p = 0.411, p = 0.341, respectively).

**Table 2:** Comparisons of the clinical and laboratory characteristics of the controlled, uncontrolled, and healthy control groups.

	Controlled(n=12)	Uncontrolled(n=22)	Control group (n=31)	p
Age (years)	9.6 (9.7)	10.8 (4.7)	10.5 (6.3)	0.854a
Sex (M/F)	6/6	11 / 11	15/16	0.992b
Puberty (Prepubertal/pubertal)	7/6	9 / 12	11/20	0.330b
Normal/overweight/obese (n)	7/3/3	8/5/8	28/1/2	<0.001b
SW/SV	9/3	13 / 9	-	0.354b
HV SDS	–0.67 (1.58)	–0.35 (4.49 )	-	0.461c
BA/CA ratio	1.08 (0.15)	1.2 (0.30)	-	0.075c
Hydrocortisone dose (mg/m2/day)	12.5 ± 4.8	13.0 ± 3.7	-	0.759d
Fludrocortisone (mg/m2/day)	0.10 (0.14)	0.08 (0.04)	-	0.186c
BMI SDS	1.04 ± 0.6	1.31 ± 0.7	–0.34 ± 1.3	<0.001e
SBP SDS	0.36 (1.7)	0.0 (1.2)	–0.42 (0.9)	0.329a
DBP SDS	0.55 ± 1.0	0.20 ± 0.8	0.48 ± 0.6	0.533e
FBG (mg/dL)	83.5 (14.7)	84.5 (13.0)	84.5 (13.0)	0.763a
HDL (mg/dL)	56.6 ± 10.8	57.6 ± 9.1	52.1 ± 9.6	0.196e
LDL (mg/dL)	102.4 ± 32.9	98.7 ± 29.7	95.5 ± 28.8	0.576e
TC (mg/dL)	175.2 ± 33.5	174.9 ± 33.8	161.5 ± 33.1	0.140e
TG (mg/dL)	74.5 (34.8)	75.0 (49.8)	79.0 (49.2)	0,810a
17- OH progesterone (ng/mL)	2.2 (5.8)	17.0 (24.4)	-	<0.001c
Androstenedione ng/mL	0.3 (1.2)	1.6 (7.3)	-	0.012c
CIMT (mm)	0.45 (0.03)	0.47 (0.04)	0.47 (0.07)	0.127a

aKruskal–Wallis test, bChi-squares test, cMann–Whitney U test, dStudent’s t-test, eOne way ANOVA test. The values are presented as mean ± SD or median (IQR). M: male, F: female, SW: salt wasting, SV: simple virilizing, HV SDS: Height velocity standard deviation score SDS, BA: Bone age, CA: Chronological age, BMI-SDS: Standard deviation score of body mass index, SBP: Systolic blood pressure, DBP: Diastolic blood pressure, FBG: Fasting blood glucose, HDL: High-density lipoprotein, LDL: Low-density lipoprotein, TC: total cholesterol, TG: triglyceride, CIMT: Carotid intima-media thickness.

There was no correlation between CIMT and age, weight SDS, height SDS, BMI SDS, hydrocortisone dose, fludrocortisone dose, SBP SDS, DBP SDS, FBG, HDL, LDL, TC, TG, 17-OH progesterone, or AS levels (p > 0.05) (data not shown). Blood pressure (SDB SDS, DBP SDS) was not correlated with hydrocortisone dose, fludrocortisone dose, and duration of treatment (data not shown). 

## 5. Discussion

Carotid intima-media thickness is an imaging technique used to predict subclinical atherosclerosis, which is an early predictor of future cardiovascular disorders [12]. It has been reported that endothelial dysfunction was associated with CIMT progression [19]. Carotid intima-media thickness has been reported to be associated with family history, ethnicity, advanced age, male sex, high body fat, high blood pressure, presence of diabetes or glucose intolerance, and high serum cholesterol and triglyceride levels [20]. It has been reported that obesity, hypertension, dyslipidemia, insulin resistance and hypercortisolism or hyperandrogenism may cause vascular dysfunction in patients with CAH, resulting in coronary artery disease [21]. Conflicting data have been reported on the effects of CAH on CIMT. Some studies on subclinical atherosclerosis have reported that CIMT was increased in children and adult patients with CAH [11,12,15,22–25]. Nonetheless, Harrington et al. [19] compared patients with obesity and CAH to healthy subjects in terms of CIMT and found no difference between the groups. In another study, Kim et al. [13] reported that CIMT values were similar between CAH and healthy subjects, but significantly higher in obese subjects compared to nonobese subjects in both the patient and control groups. In the same study, it has been shown that CIMT values in male CAH cases were significantly higher than in females. In subgroup analysis, CIMT values have been reported to be similar in SW and SV type CAH cases [12,13]. In the study by Metwalley et al. [22], CIMT values have been found to be significantly higher in uncontrolled CAH cases than in well-controlled cases and in both male and female CAH cases compared to healthy subjects. In the same study, it has been shown that CIMT was related to age, duration of treatment, equivalent of hydrocortisone dose, 17-hydroxyprogesterone, and testosterone. In our study, CIMT values were similar between CAH patients and healthy subjects. In addition, CIMT values were found to be similar in subgroups according to sex, CAH forms (SW and SV) and control status (controlled/uncontrolled) compared to healthy subjects. In addition, in our study, no correlation was found between CIMT and age, BMI, hydrocortisone dose, 17-hydroxyprogesterone or androstenedione, conflicting with the findings of some studies [13,22]. However, these findings do not suggest an increased risk of cardiovascular disease in our CAH patients at the age when they were evaluated. The reasons for the similarity in CIMT values between CAH cases and healthy subjects in the current study may be due to i) the low number of cases, ii) failure to evaluate the familial characteristics of patients or healthy subjects in terms of risk of cardiovascular disease, iii) lack of time for vascular change to occur for CIMT increase to be observed. 

The prevalence of obesity, hypertension, dyslipidemia, and insulin resistance have previously been reported to be increased in CAH patients. In our study, the height SDS was lower, and BMI SDS was higher in patients with CAH compared to healthy subjects, consistent with the literature [26,27]. In addition, in our study, 32.3% of the CAH patients were obese. Similarly, increased obesity rates (16.8%–25%) have previously been reported [5,12,15]. Obesity in CAH patients has been associated with glucocorticoid therapy, high androgen levels, leptin resistance, and decreased lipolysis due to the reduction in catecholamine secretion [21]. 

Increased vascular tone due to obesity, hyperinsulinemia, and glucocorticoid treatment may cause hypertension in CAH patients [6]. Hypertension is a major cardiovascular morbidity in children and adolescents with CAH [5]. In our study, none of the children in the CAH group had hypertension. Moreover, in our study, no difference was found between the CAH patients and healthy subjects in terms of SBP or DBP. Some studies have reported normal resting blood pressure values [13,22,25,28] and 24-h BP profile [29] in children and young adults with CAH compared to healthy subjects, similar to our results. On the other hand, in studies performing blood pressure assessment by 24-h BP profile, Da Silva et al. [30] reported day-time SBP increase (between 90 and 95 percentile range) in 5 of 11 cases with CAH. Völkl et al. [31] reported significantly higher SBP SDSs than healthy subjects in both day-time and night-time, and Roche et al. [4] reported 58% systolic and 24% diastolic hypertension in 38 SW type CAH cases. Interestingly, in CAH cases with normal resting SBP and DBP, peak SBP and DBP values and increases in SBP and DBP values have been found to be significantly higher during exercise than resting or daily routine activity [32]. Although BP profile is not affected in daily routine activities, it seems that BP responses may be impaired during exercise in CAH cases due to their metabolic dysregulation. Finally, contradictory results from different studies could be explained by the facts that a) hypertension may have been missed due to the lack of 24-h blood pressure monitoring in CAH patients b) significant alterations in vascular structure may not yet have developed, and c) impaired exercise performance, which may be an early marker of cardiovascular disease in CAH patients, was not evaluated.

Increased frequency of obesity, insulin resistance, hydrocortisone treatment and high androgen exposure is expected to deteriorate the lipid profiles of CAH cases. However, contrary to this expectation, the lipid profiles were similar to healthy controls in this study. Most studies on children and adults have reported similar lipid profiles in CAH cases and healthy control subjects, similar to our study [11,12,24,33–35]. In their study on prepubertal CAH cases, Botero et al. [36] reported that TC, LDL, and HDL values were similar, but TG values were significantly higher in CAH cases compared to healthy subjects. However, it has been noted that prednisone, which increases the risk of metabolic alteration more than hydrocortisone, was used in treatment in this study. In addition, Zimmermann et al. [37] reported higher small, dense LDL and lower HDL in CAH cases. However, their study groups were quite heterogeneous in terms of age (age range: 4–31), which was one of the main limitations of the study. One study suggested that dyslipidemia may take time to develop, which could be the reason for the lack of changes in lipid profile in children with CAH [21]. In contrast to this hypothesis, Falhammar et al. [35] reported similar serum TC, HDL, LDL, and TG levels between CAH patients aged <30 years and CAH patients aged >30 years. Furthermore, in the same study, the HDL/LDL ratio, which is considered to be protective against cardiovascular disease, has been reported to be significantly higher in CAH patients >30 years of age. In conclusion, contrary to expectations, the current study showed no evidence to support lipid alteration in CAH, as in many other studies. However, the low number of cases in both our study and the previous studies may have prevented us from demonstrating the differences in lipid profiles.

The main limitation of our study was that we could not exclude the impact of blood pressure changes. Although there was no difference between the groups in terms of blood pressures, intermittent elevated blood pressure may cause endothelial dysfunction in the congenital adrenal hyperplasia group [32]. Therefore, it would be more appropriate to perform 24-h blood pressure monitoring in congenital adrenal hyperplasia patients. Another limitation was the low number of subgroup cases. In addition, 17-hydroxyprogesterone and androstenedione levels, which are indirect indicators of increase in androgens, were evaluated, but testosterone and DHEAS levels could not be measured. Therefore, we could not investigate whether androgen levels were correlated with CIMT. The effect of genetic factors affecting CIMT and cardiovascular disease risk was not considered in this study, as well. The risk of cardiovascular disease is associated with fat distribution. The risk of this disease is determined by waist circumference regardless of obesity and is more reliable than BMI [38]. We have not performed waist circumference measurements. 17-OH progesterone was not studied in the control group. These are other limitations in this study. The last limitation of the current study is that androstenedione and 17-OH progesterone levels were studied with the radioimmunoassay method. In recent studies, it has obviously been demonstrated that liquid chromatography-tandem mass spectrometry is more reliable in steroid hormones, precursors, and metabolic parameters than the RIA method [39].

In conclusion, in the present study, the CIMT values of CAH cases were found to be similar to those of healthy subjects. In addition, CIMT levels were similar according to sex, disease control status, and CAH type and were not associated with any anthropometric or metabolic parameters. Although these findings suggest that the risk of cardiovascular disease is low in CAH patients during childhood and adolescence, it should be kept in mind that clinically significant endothelial dysfunction may not yet have occurred due to early age.

## Funding

This research did not receive any specific grant from any funding agency in the public, commercial or not-for-profit sector.
